# Supplementary material for: Genetic variation in polyploid forage grass: Assessing the molecular genetic variability in the Paspalum genus
Source: BMC Genet. 2013 Jun 8;14:50. doi: 10.1186/1471-2156-14-50 (PMC3682885; doi:10.1186/1471-2156-14-50)
Supplement: Additional file 2 — Paspalum notatumaccessions evaluated in this study.Paspalum notatum accessions evaluated in this study. The CODE used in the figures, ID, collector identification, BRA CODE, site of origin, chromosome number, ploidy level, geographic coordinates and availability of morphological data are shown. [file 1471-2156-14-50-S2.docx]

## Additional File 2 - Paspalum notatum accessions evaluated in this study

| CODE | ID | COLLECTOR | BRA CODE | ORIGIN | CN | | | PL | | LAT | | LONG | | MDA | |
| --- | --- | --- | --- | --- | --- | --- | --- | --- | --- | --- | --- | --- | --- | --- | --- |
| 1 | 49 | V 14329 | BRA - 021563 | Capivari do Sul (RS), Brazil | 2n=40 | | | 2n=4X | | -30.13 | | -50.57 | | Yes | |
| 2 | 59 | V 14327 | BRA - 021547 | Capivari do Sul (RS), Brazil | 2n=40 | | | 2n=4X | | -30.12 | | -50.55 | | Yes | |
| 3 | M7 | V14327 | NA | Capivari do Sul (RS), Brazil | 2n=40 | | | 2n=4X | | -30.17 | | -50.55 | | Yes | |
| 4 | M6 | V14329 | NA | Capivari do Sul (RS), Brazil | 2n=40 | | | 2n=4X | | -30.13 | | -50.57 | | Yes | |
| 5 | M17 | V14870 | NA | Capivari do Sul (RS), Brazil | 2n=40 | | | 2n=4X | | -30.20 | | -50.50 | | Yes | |
| 6 | M18 | V14871 | NA | Capivari do Sul (RS), Brazil | 2n=40 | | | 2n=4X | | -30.20 | | -50.50 | | Yes | |
| 7 | M5 | V14326 | NA | Capivari do Sul (RS), Brazil | 2n=40 | | | 2n=4X | | -30.20 | | -50.50 | | Yes | |
| 8 | M14 | V14866 | NA | Capivari do Sul (RS), Brazil | 2n=40 | | | 2n=4X | | -30.20 | | -50.50 | | Yes | |
| 9 | M49 | MD s/n | NA | Mostardas (RS), Brazil | 2n=40 | | | 2n=4X | | -30.80 | | -50.70 | | Yes | |
| 10 | M25 | MD s/n | NA | Lavras do Sul (RS), Brazil | 2n=40 | | | 2n=4X | | -30.90 | | -54.00 | | Yes | |
| 11 | M26 | MD s/n | NA | Caçapava do Sul (RS), Brazil | 2n=40 | | | 2n=4X | | -30.50 | | -53.50 | | Yes | |
| 12 | M27 | MD s/n | NA | Lavras do Sul (RS), Brazil | 2n=40 | | | 2n=4X | | -30.90 | | -54.00 | | Yes | |
| 13 | M28 | MD s/n | NA | Caçapava do Sul (RS), Brazil | 2n=40 | | | 2n=4X | | -30.50 | | -53.50 | | Yes | |
| 14 | M23 | MD s/n | NA | Encruzilhada do Sul (RS), Brazil | 2n=40 | | | 2n=4X | | -30.60 | | -52.60 | | Yes | |
| 15 | M24 | MD s/n | NA | Encruzilhada do Sul (RS), Brazil | 2n=40 | | | 2n=4X | | -30.60 | | -52.60 | | Yes | |
| 16 | M33 | MD s/n | NA | Vacaria (RS), Brazil | 2n=40 | | | 2n=4X | | -28.50 | | -51.00 | | Yes | |
| 17 | M30 | MD s/n | NA | Bagé (RS), Brazil | 2n=40 | | | 2n=4X | | -31.30 | | -54.10 | | Yes | |
| 18 | M8 | V14614 | NA | Japorã (MS), Brazil | 2n=40 | | | 2n=4X | | -23.75 | | -54.10 | | Yes | |
| 19 | 62 | V 14614 | BRA - 023566 | Japorã (MS), Brazil | 2n=40 | | | 2n=4X | | -23.75 | | -54.00 | | Yes | |
| 20 | 65 | Lr 1 | BRA - 019178 | São Carlos (SP), Brazil | NA | | | NA | | -22.02 | | -47.88 | | No | |
| 21 | 64 | V 11664 | BRA - 012254 | Macapá (AP), Brazil | NA | | | NA | | 0.02 | | -51.05 | | No | |
| 22 | M50 | CN s/n | NA | Piracicaba (SP), Brazil | 2n=40 | | | 2n=4X | | -22.70 | | -47.90 | | Yes | |
| 23 | M32 | MD s/n | NA | Barretos (SP), Brazil | 2n=40 | | | 2n=4X | | -20.50 | | -48.60 | | Yes | |
| 24 | M10 | V14287 | NA | Candói (PR), Brazil | 2n=40 | | | 2n=4X | | -25.50 | | -52.00 | | Yes | |
| 25 | M11 | V14282 | NA | Candói (PR), Brazil | 2n=40 | | | 2n=4X | | -25.50 | | -52.00 | | Yes | |
| 26 | M35 | MD s/n | NA | São Borja (RS), Brazil | 2n=40 | | | 2n=4X | | -28.70 | | -55.90 | | Yes | |
| 27 | M31 | MD s/n | NA | André da Rocha (RS), Brazil | 2n=40 | | | 2n=4X | | -28.60 | | -51.50 | | Yes | |
| 28 | M29 | MD s/n | NA | Lavras do Sul (RS), Brazil | 2n=40 | | | 2n=4X | | -30.90 | | -54.00 | | Yes | |
| 29 | M4 | V14310 | NA | Barra do Quaraí (RS), Brazil | 2n=40 | | | 2n=4X | | -30.10 | | -57.40 | | Yes | |
| 30 | 61 | V 14613 | BRA - 023558 | Japorã (MS), Brazil | NA | | | NA | | -23.48 | | -54.00 | | No | |
| 31 | 60 | V 14600 | BRA - 023523 | Coronel Sapucaia (MS), Brazil | NA | | | NA | | -23.35 | | -55.52 | | No | |
| 32 | 57 | Ip 6925 | BRA - 001074 | Paraguay | NA | | | NA | | -25.25 | | -57.67 | | Yes | |
| 33 | 56 | Ip 6915 | BRA - 001112 | Pensacola (FL) U.S. | NA | | | NA | | 30.50 | | -82.17 | | Yes | |
| 34 | 53 | EEA 673 | BRA - 007986 | Eldorado do Sul (RS), Brazil | 2n=40 | | | 2n=4X | | -30.10 | | -51.58 | | Yes | |
| 35 | 48 | V 9782 | BRA - 006513 | Uruguaiana (RS), Brazil | 2n=40 | | | 2n=4X | | -29.55 | | -56.78 | | Yes | |
| 36 | 52 | V 14658 | BRA - 023728 | Costa Rica (MS), Brazil | NA | | | NA | | -18.56 | | -53.13 | | No | |
| 37 | M2 | V14244"B" | NA | Uruguaiana (RS), Brazil | 2n=40 | | | 2n=4X | | -29.70 | | -57.00 | | Yes | |
| 38 | M51 | CN s/n | NA | Posadas, Argentina | 2n=40 | | | 2n=4X | | -27.38 | | -55.88 | | Yes | |
| 39 | M54 | CN s/n | NA | Eldorado do Sul (RS), Brazil | 2n=40 | | | 2n=4X | | -30.10 | | -51.60 | | Yes | |
| 40 | M38 | CN s/n | NA | Santo Tome, Argentina | 2n=40 | | | 2n=4X | | -28.60 | | -56.05 | | Yes | |
| 41 | M46 | CN s/n | NA | Santo Tome, Argentina | 2n=40 | | | 2n=4X | | -28.60 | | -56.05 | | Yes | |
| 42 | M37 | CN s/n | NA | Santo Tome, Argentina | 2n=40 | | | 2n=4X | | -28.60 | | -56.05 | | Yes | |
| 43 | M39 | CN s/n | NA | Santo Tome, Argentina | 2n=40 | | | 2n=4X | | -28.60 | | -56.05 | | Yes | |
| 44 | M42 | CN s/n | NA | Santo Tome, Argentina | 2n=40 | | | 2n=4X | | -28.60 | | -56.05 | | Yes | |
| 45 | M44 | CN s/n | NA | Santo Tome, Argentina | 2n=40 | | | 2n=4X | | -28.60 | | -56.05 | | Yes | |
| 46 | M36 | CN s/n | NA | Santo Tome, Argentina | 2n=40 | | | 2n=4X | | -28.60 | | -56.05 | | Yes | |
| 47 | M41 | CN s/n | NA | Santo Tome, Argentina | 2n=40 | | | 2n=4X | | -28.60 | | -56.05 | | Yes | |
| 48 | M56 | V14931 | NA | Alegrete (RS), Brazil | 2n=40 | | | 2n=4X | | -29.80 | | -55.80 | | Yes | |
| 49 | M66 | MD s/n | NA | Colonia, Uruguay | 2n=40 | | | 2n=4X | | -34.14 | | -57.46 | | Yes | |
| 50 | M67 | MD s/n | NA | Colonia, Uruguay | 2n=40 | | | 2n=4X | | -34.14 | | -57.46 | | Yes | |
| 51 | M55 | V14921 | NA | Quaraí (RS), Brazil | 2n=40 | | | 2n=4X | | -30.40 | | -56.40 | | Yes | |
| 52 | 55 | V 9684 | BRA - 006301 | Uruguaiana (RS), Brazil | 2n=40 | | | 2n=4X | | -29.71 | | -57.07 | | Yes | |
| 53 | 54 | V 9747 | BRA - 006467 | Alegrete (RS), Brazil | NA | | | NA | | -29.08 | | -55.58 | | Yes | |
| 54 | Pensacola | St s/n | NA | Viamão (RS), Brazil | 2n=20 | | | 2n=2X | | -30.00 | | -50.80 | | Yes | |
| 55 | 66 | Q 3667 | BRA - 019470 | Corrientes, Argentina | NA | | | NA | | -27.58 | | -58.75 | | No | |
| 56 | 58 | V 14829 | BRA - 024236 | Candói (PR), Brazil | 2n=20 | | | 2n=2X | | -25.45 | | -51.87 | | No | |
| 57 | 63 | V 9607 | BRA - 006173 | Bagé (RS), Brazil | 2n=20 | | | 2n=2X | | -31.30 | | -54.08 | | Yes | |
| *CN=Chromosome number; PL=ploidy level; MDA=morphological data available;  LAT=latitude (decimal degrees); LONG=longitude (decimal degrees). | | | | | |  |  | |  | |  | |  | |  |
