# Supplementary figures and images for: Genetic variation in polyploid forage grass: Assessing the molecular genetic variability in the Paspalum genus
Source: BMC Genet. 2013 Jun 8;14:50. doi: 10.1186/1471-2156-14-50 (PMC3682885; doi:10.1186/1471-2156-14-50)

$$\text{DeltaK} = \text{mean}(|L''(K)|) / \text{sd}(L(K))$$

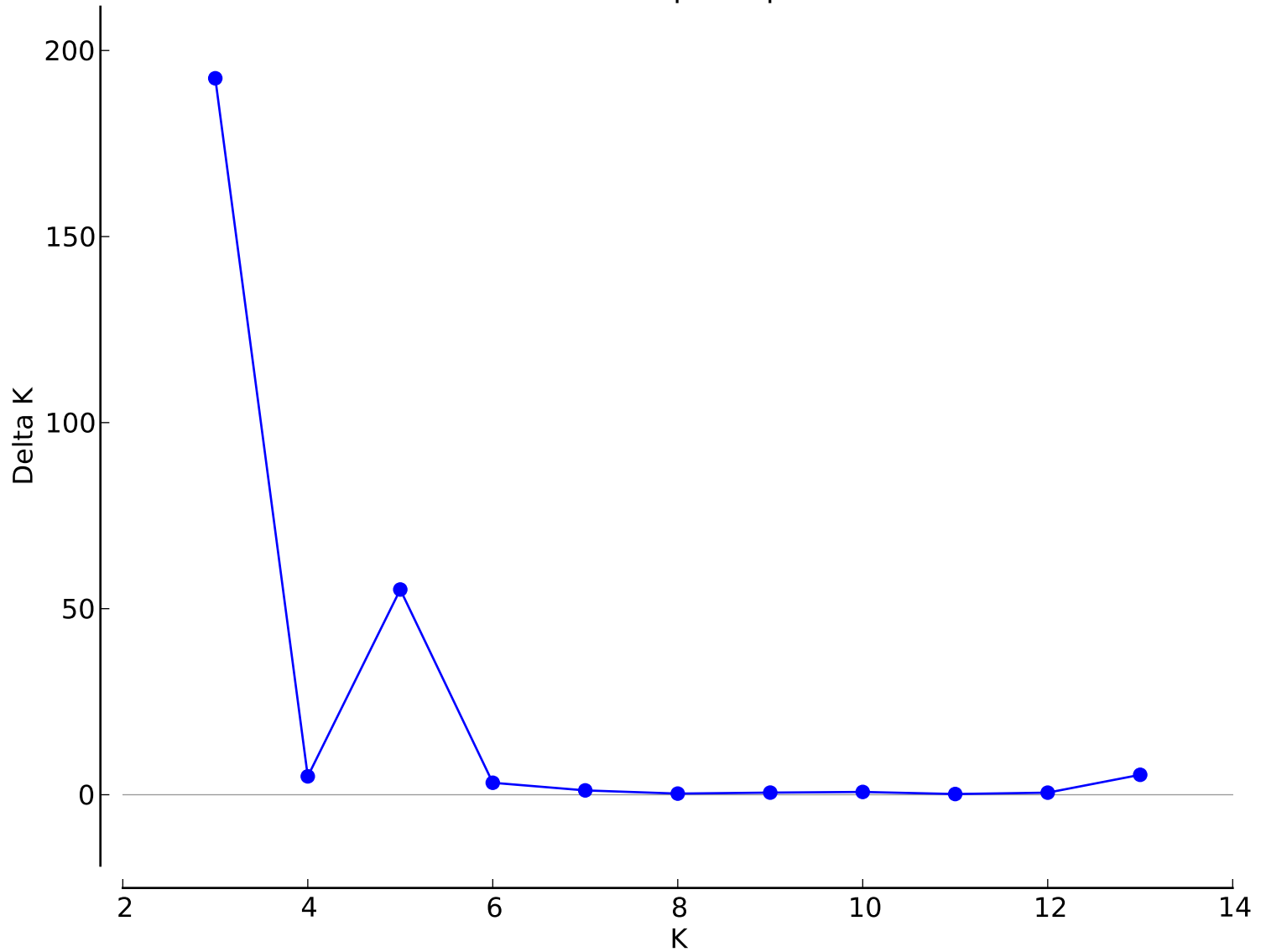

Supplement: Additional file 5 — Magnitude ofΔK from STRUCTURE analysis of the germplasm. Magnitude of ΔK from STRUCTURE analysis of K (arithmetic mean ± s.d. over 10 replicates) calculated following the ΔK method proposed by Evanno et al. [84] for Paspalum microsatellite data. The modal values of these distributions indicate that the true K, or the uppermost level of the STRUCTURE analysis, is seven genetic groups. [file 1471-2156-14-50-S5.pdf]

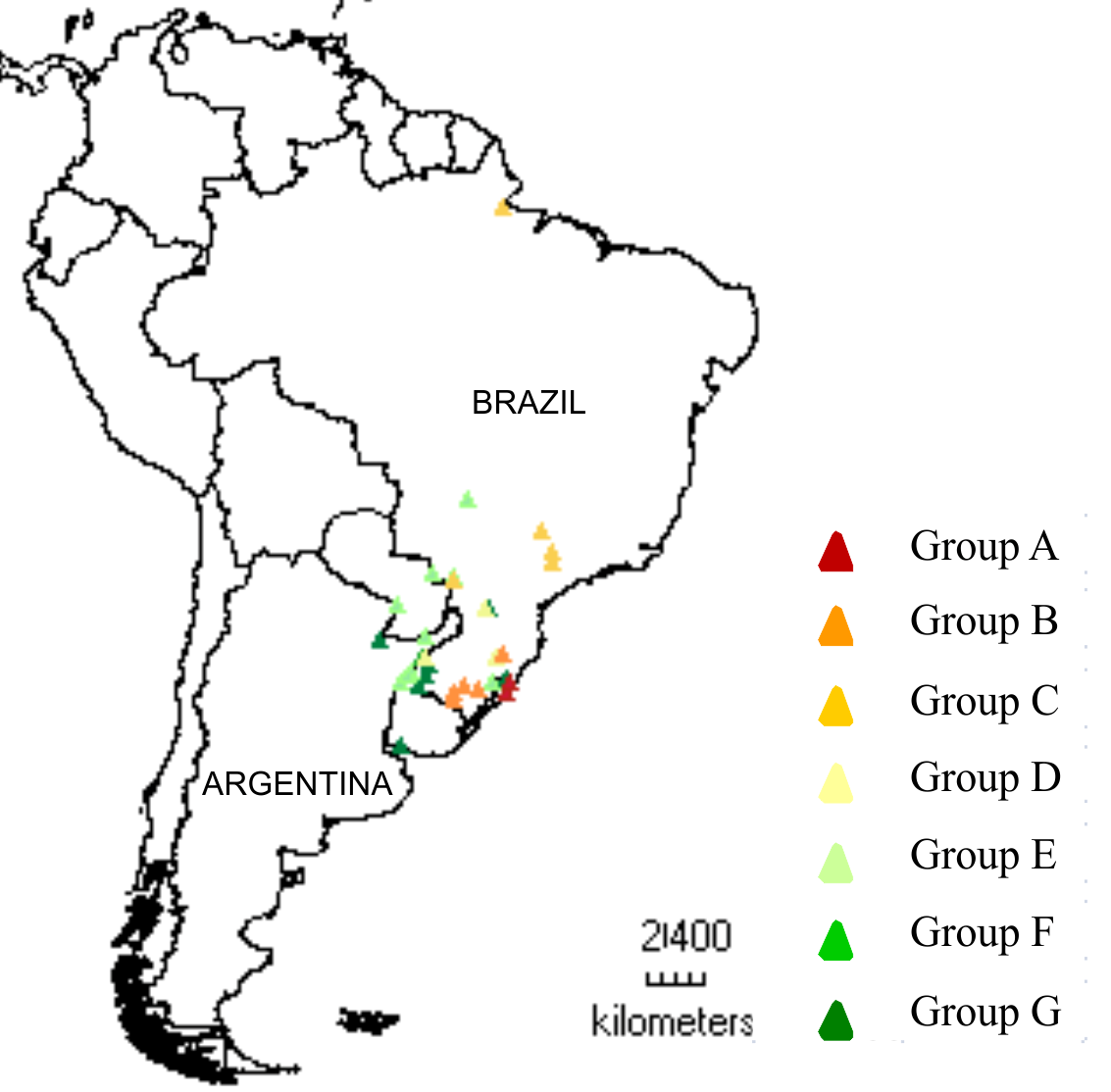

Supplement: Additional file 11 — Map of the geographic distribution of 57 of thePaspalum notatumaccessions analyzed. Geographic distribution of the 57 Paspalum notatum accessions that were analyzed genotypically and phenotypically. Point colors correspond to the colors in the STRUCTURE clusters from Figure 5. A sole accession from Florida, USA, has not been represented in the map. [file 1471-2156-14-50-S11.tiff]
