# Supplementary material for: Genetic variation in polyploid forage grass: Assessing the molecular genetic variability in the Paspalum genus
Source: BMC Genet. 2013 Jun 8;14:50. doi: 10.1186/1471-2156-14-50 (PMC3682885; doi:10.1186/1471-2156-14-50)
Supplement: Additional file 7 — Mean probabilities for each population from the STRUCTURE analysis for K = 7 forPaspalum notatum. Mean probabilities for each population from the STRUCTURE analysis for K = 7 for Paspalum notatum. [file 1471-2156-14-50-S7.docx]

**Additional File 7 – Mean probabilities for each population from the STRUCTURE analysis for K=7 for *Paspalum notatum.***

| Botanical Variety | A | B | C | D | E | F | G | P<0.5 |
| --- | --- | --- | --- | --- | --- | --- | --- | --- |
| *P. notatum* var. *notatum* | **9/**0.32 | **8**/0.25 | **6/**0.20 | **5/**0.20 | 0.00 | 0.02 | 0.01 | 0 |
| *P. notatum* var. *latiflorum* | 0.01 | 0.01 | 0.02 | 0.06 | **3/**0.15 | **8/**0.43 | **6/**0.33 | 1 |
| *P. notatum* var. *saurae* | 0.01 | 0.00 | 0.00 | 0.01 | 0.01 | 0.01 | **4**/0.96 | 0 |
| Without variety identification | 0.02 | 0.00 | 0.00 | 0.02 | **7/**0.93 | 0.02 | 0.01 | 0 |

The numbers in bold refer to the number of individuals assigned to one of the seven groups with a probability greater than 0.50. The last line refers to the remaining individuals, which may have been assigned to other groups.
